# 

**Published:** 1906-03

**Authors:** 


### THE BRISTOL

### Medico-Chirurgical Journal.

47

A JOURNAL OF THE MEDICAL SCIENCES FOR THE WEST OF ENGLAND AND SOUTH WALES

*PUBLISHED UNDER THE AUSPICES OF*

*THE BRISTOL MEDICO-CHIRURGICAL SOCIETY.*

EDITOR:
R. SHINGLETON SMITH, M.D., B.Sc.,

WITH WHOM ARE ASSOCIATED
J. MICHELL CLARKE, M.A., M.D., J. LACY FIRTH, M.S., M.D., AND JAMES SWAIN, M.S., M.D.

ASSISTANT-EDITOR:
P. WATSON WILLIAMS, M.D.

EDITORIAL SECRETARY:
JAMES TAYLOR

"Scire est nescire, nisi id me

Scire alius sciret."

LIBRARY

SURGEON GENERAL'S OFFICE

AUG. 21. 1907

197249.

*VOL. XXIV*

BRISTOL: J. W. ARROWSMITH.
LONDON: J. & A. CHURCHILL, 7 GREAT MARLBOROUGH STREET.
1906.

WI
BR323

J. W. Arrowsmith

Publisher · Bristol

33756

### CONTENTS OF VOLUME XXIV.

| | Page |
|---|---|
| Anti-Tuberculous Vaccines and Sera. By J. M. FORTESCUE-BRICKDALE, M.A., M.D.Oxon. ... ... ... ... ... | 1 |
| The Prognosis of Pulmonary Tuberculosis. By J. J. S. LUCAS, M.D., B.A.Lond. ... ... ... ... ... ... ... ... | 12 |
| Why Defective Nasal Respiration Impedes Growth and Development. By P. WATSON WILLIAMS, M.D.Lond. ... ... ... ... | 17 |
| Some Effects of Fright in Medicine. By J. R. CHARLES, M.A., M.D.Camb., M.R.C.P. ... ... ... ... ... ... | 23 |
| Colitis Polyposa. By CAREY COOMBS, M.D., B.S.Lond. ... ... | 31 |
| Notes of a Case of Acute Yellow Atrophy of the Liver. By F. H. EDGEWORTH, M.B.Cantab., D.Sc.Lond. ... ... ... | 37 |
| A Case of Acute Yellow Atrophy of the Liver in a Child. By BERTRAM M. H. ROGERS, B.A., M.D., B. Ch.Oxon. ... ... | 40 |
| Notes on a Case of Splenomegalic Biliary Cirrhosis in a Boy aged six years. By EDWARD CECIL WILLIAMS, M.B.Cantab. ... | 42 |
| Clinical Note on a Case of Epithelioma of Tongue in a Young Woman. By C. HAMILTON WHITEFORD, M.R.C.S., L.R.C.P.... | 45 |
| The Relation of the Pelvic Floor to Pelvic Displacements and Pain in the Female. By ERNEST W. HEY GROVES, M.D., M.S.Lond. F.R.C.S. ... ... ... ... ... ... ... ... ... | 97 |
| Lumbago. By CHARLES E. S. FLEMMING, M.R.C.S., L.R.C.P. ... | 110 |
| Difficulties in the Surgical Diagnosis and Treatment of Cases associated with Vomiting in Children. By JAMES SWAIN, M.S., M.D.Lond., F.R.C.S. ... ... ... ... ... ... ... | 116 |
| New Methods of Clinical Pathology. By I. WALKER HALL, M.D.Vict. | 121 |
| Tubal Pregnancy—its Diagnosis and Treatment. By C. HAMILTON WHITEFORD, M.R.C.S., L.R.C.P. ... ... ... ... ... ... | 126 |
| The New Theory and Prophylaxis of Puerperal Eclampsia. By ELIZA L. WALKER DUNBAR, M.D.Zurich ... ... ... ... ... | 132 |
| Health Notes in Bristol, 1905. By D. S. DAVIES, M.D.Lond. ... | 137 |

CONTENTS.

Page

The Adaptation of Means in the Prevention of Disease. By D. S. Davies, M.D.Lond., D.P.H.Cantab. ... ... ... 193

The Treatment of Tabetic Ataxia by Methodical Exercises. By Maurice Faure, M.D. ... ... ... ... ... ... 210

A Case of Mesenteric Thrombosis. By G. Munro Smith, M.R.C.S. 216

On the Treatment of Diabetes with Secretin. By J. R. Charles, M.A., M.D.Cantab., M.R.C.P. ... ... ... ... ... 218

The Doctor as Expert and Pioneer. By Charles J. Whitby, M.D. 222

The Uses of Static Electricity as an Aid to Recovery. By Preston King, M.A., M.D.Cantab. ... ... ... ... ... ... 233

The Explanation of Alveolar Cleft Palate. By Edward Fawcett, M.D.Edin. ... ... ... ... ... ... ... ... 237

X-Ray Therapeutics. By James Taylor, M.R.C.S. ... ... ... 289

Fibroma of the Abdominal Wall. By J. Lacy Firth, M.S. Lond., F.R.C.S. ... ... ... ... ... ... ... ... ... 304

The Preservation of the Limb in the Treatment of Sarcoma of Bone. By Charles A. Morton, F.R.C.S. ... ... ... ... ... 308

Proteins and Protein Metabolism. By J. M. Fortescue-Brickdale, M.A., M.D.Oxon. ... ... ... ... ... ... ... 318

The Long Fox Lecture. By Alfred J. Harrison, M.B.Lond. ... 325

Progress of the Medical Sciences ... ... 47, 140, 249, 344

Reviews of Books ... ... ... ... ... 61, 157, 261, 359

Editorial Notes ... ... ... ... ... 70, 171, 268, 370

Notes on Preparations for the Sick ... 81, 179, 281, 376

The Library of the Bristol Medico-Chirurgical Society ... ... ... ... ... ... 85, 185, 284, 379

Meetings of Societies ... ... ... ... ... 88, 188, 382

Obituary Notices ... ... ... ... ... 93, 191, 286, 386

Local Medical Notes ... ... ... ... 95, 192, 287, 391

### The Library of the Bristol Medico-Chirurgical Society.

*The following donations have been received since the publication of the List in December:*

*February 28th, 1906.*

| | |
|---|---|
| L. M. Griffiths (1) | 7 volumes. |
| J. H. Loch, M.D. (2) | 2 ,, |
| The Medical Officer of Health of the London County Council (3) | 1 volume. |
| The Staff of Middlesex Hospital (4) | 1 ,, |
| The Council of the Otological Society (5) | 1 ,, |
| Pennsylvania State Medical Society (6) | 1 ,, |
| The Royal Medical and Chirurgical Society (7) | 1 ,, |
| R. Shingleton Smith, M.D. (8) | 11 volumes. |
| Scholastic Trading Company (9) | 2 ,, |

### FIFTY-NINTH LIST OF BOOKS.

The titles of books mentioned in previous lists are not repeated.

The figures in brackets refer to the figures after the names of the donors, and show by whom the volumes were presented. The books to which no such figures are attached have either been bought from the Library Fund or received through the *Journal*.

**Allbutt, T. C.** ... *On Professional Education* ... ... ... ... ... ... ... 1906
**Allbutt and H. D. Rolleston, T. C.** [Eds.] *A System of Medicine.* Vol. I. 2nd Ed. 1905
**Allchin, W. H.**... *The Harveian Oration* ... ... ... ... ... ... ... ... (8) 1903
**Andrews, C. Beck and H.** *Treatise on Photographic Lenses* ... ... (8) [N.D.]
**Ashby and G. A. Wright, H.** *The Diseases of Children* ... ... 5th Ed. 1905
**Beck and H. Andrews, C.** *Treatise on Photographic Lenses* ... ... (8) [N.D]
**Beevor, C. E.** ... *The Croonian Lectures, 1903* ... ... ... ... ... ... (8) 1904
**Bell, B.** ... ... *A Treatise on the Hydrocele* ... ... ... ... ... ... (1) 1794
**Bell, J.** ... ... *Discourses on the Nature and Cure of Wounds.* 3rd Ed. (1) 1812
*Bibliographique universel, Manuel abrégé du Répertoire* ... ... ... .. ... 1905
**Brinckman, A.** ... *Notes on the Care of the Sick* ... ... ... ... ... ... (1) 1879
**Bulkley, L. D.** ... *Relations of Skin Diseases to Internal Disorders* ... ... 1906
*Catalogue of the Library of the Royal Medical and Chirurgical Society.* Supplement IX.... ... ... ... ... ... ... ... ... ... ... (7) 1901
**Chance, E. J.** ... *Bodily Deformities.* (Ed. by J. Poland.) Vol. I. 2nd Ed. 1905
**Chapman, W. L.**... *The Sequelæ of Gonorrhœa in both Sexes* ... ... ... 1905
**Church, Sir W. S.** *Presidential Address to the Royal College of Physicians* (8) 1904
**Edmunds, W.** ... *Pathology and Diseases of the Thyroid Gland*... ... (8) 1901
*Egypt, Health and Pleasure in* ... ... ... ... ... ... ... ... ... ... ... (8) [N.D.]
**Giles, A. E.** ... *Gynæcological Diagnosis* ... ... ... ... ... ... ... ... 1906
**Goodhart, J. F.**... *The Diseases of Children.* (Ed. by G. F. Still.) 8th Ed. 1905
**Gould, G. M.** ... *Biographic Clinics.* Vol. III. ... ... ... ... ... ... 1905
**Gubb, A. S.** ... *Algiers as a Winter Resort* ... ... ... ... ... ... (8) 1905
**Haegler, C. S.** ... *The Cleansing, Disinfection and Protection of the Hands.* (Tr. by C. H. Watson) ... ... ... ... ... ... ... 1906
"*Hazell's Annual*" *Guide to the New House of Commons* ... ... 2nd Ed. 1906
**Huggard, W. R.** *A Handbook of Climatic Treatment*... ... ... ... ... ... 1906
**Janeway, T. C.**... *The Clinical Study of Blood Pressure* ... ... ... ... 1904
**Liebreich, R.** ... *Atlas der Ophthalmoscopie* ... ... ... ... ... ... (2) 1863
**Mackenzie, S. C.** *Medico-Legal Experience in Calcutta* ... .. ... (2) 1891
*Orotava as a Health Resort*... ... ... ... ... ... ... ... ... ... ... ... (8) [N.D.]
**Rolleston, T. C. Allbutt and H. D.** [Eds.] *A System of Medicine.* Vol. I. 2nd Ed. 1905
*Royal (The) Medical and Chirurgical Society of London, Centenary 1805—1905* 1905
**Shelly, C. E.** ... *A Preliminary Inquiry concerning the Milk Supply of Schools* ... ... ... ... ... ... ... ... ... ... 1906
**Smee, A. H.** ... *Suggestions as to Lines for Future Research* ... ... (1) 1881
**Stewart, G. N.** ... *A Manual of Physiology* ... ... ... ... ... ... 5th Ed. 1906
**Taylor, F.** ... ... *Some Disorders of the Spleen* ... ... ... ... ... ... (8) 1904
**Thorne, L. T.** ... *The "Nauheim" Treatment of Chronic Diseases of the Heart* .. ... ... ... ... ... ... ... 2nd Ed. 1906
**Weininger, O.** ... *Sex and Character.* (Tr. from 6th German Edition.) 1906
**Woollacott, F. J.** *The Nursing of Infectious Diseases* ... ... ... ... ... [1906]
**Wright, A. E.** ... *Anti-Typhoid Inoculation* ... ... ... ... ... ... (8) 1904
**Wright, H. Ashby and G. A.** *The Diseases of Children* ... ... 5th Ed. 1905

### TRANSACTIONS, REPORTS, JOURNALS, &c.

| | | |
|---|---|---|
| American Journal of Obstetrics, The | Vol. LII. | 1905 |
| Archives of the Middlesex Hospital | (4) Vol. VI. | 1905 |
| Bristol and Clifton Directory, Mathews's | (1) | 1853 |
| British Almanac, The | | 1906 |
| British Journal of Children's Diseases, The | Vol. II. | 1905 |
| British Journal of Dermatology, The | Vol. XVII. | 1905 |
| British Medical Journal, The | Vol. II. for | 1905 |
| Brooklyn Medical Journal, The | Vol. XIX. | 1905 |
| Catalogue of Books, The English, 1905 | | 1906 |
| Dermatological Society of Great Britain, Transactions of the | Vols. IX., X. | 1902–04 |
| Edinburgh Medical Journal, The | N.S., Vol. XVIII. | 1905 |
| Gazette des Hôpitaux de Toulouse | | 1905 |
| Glasgow Medical Journal, The | Vol. LXIV. | 1905 |
| Hazell's Annual | | 1906 |
| Hospitals—St. Bartholomew's Hospital: Statistical Tables, 1903 | (8) | 1904 |
| St. Thomas's Hospital Reports | Vol. XXXIII. | 1905 |
| Intercolonial Medical Journal of Australasia | Vol. IX. | 1904 |
| Journal of Medical Research, The | Vol. XIII. | 1904–05 |
| Journal of Tropical Medicine, The | Vol. VIII. | 1905 |
| Lancet, The | Vol. II. for | 1905 |
| London County Council, Report of Medical Officer of, for 1904 | (3) | 1905 |
| Medical Directory, The | | 1906 |
| Medical Press and Circular, The | [Vol. CXXXI.] | 1905 |
| Medical Society's Transactions, The | Vol. XXVIII. | 1905 |
| Mercy and Truth | Vol. IX. | 1905 |
| New Hampshire Medical Society, Transactions of the | | 1905 |
| Nordischen Kongresses für innere Medicin, Verhandlungen des Fünften (1904) | | 1905 |
| Ophthalmological Transactions | Vol. XXV. | 1905 |
| Otological Society, Transactions of the | (5) Vol. VI. | 1905 |
| Pennsylvania Medical Journal, The | (6) Vol. VIII. | 1905 |
| Progressive Medicine | Vol. IV. | 1905 |
| Public Health | Vol. XVII. | 1904–05 |
| Publishers' Circular, The | (9) Vols. LXXXII., LXXXIII. | 1905 |
| Scottish Medical and Surgical Journal, The | Vol. XVII. | 1905 |
| Smithsonian Report, 1904 | | 1905 |
| Society for the Study of Disease in Children, Reports of the | Vol. V. | 1904–05 |
| West London Medical Journal | Vol. X. | 1905 |
| Whitaker's Almanack | | 1906 |
| Who 's Who | | 1906 |
| Year-Book of Scientific and Learned Societies, The | | 1905 |
| Zentralblatt für Chirurgie | | 1905 |

### Local Medical Notes.

**University College, Bristol.**—I. Walker Hall, M.D., Ch.B. Vict., has been appointed Professor of Pathology. J. M. Fortescue-Brickdale, M.D., Ch.B. Oxon., has been appointed Demonstrator of Physiology. The following examination successes have recently been recorded: *M.S. Lond.:* E. W. Hey Groves. M.D., B.Sc. (University Medal). *M.B., B.S., Lond.:* H. Devine, W. J. H. Pinniger. *Group I.:* J. F. Blackett, J. B. V. Watts. *B.C. Cantab.:* F. F. Leighton, F. Shingleton Smith. *F.R.C.S., Eng.:* E. W. Hey Groves. Preliminary Scientific Examination. — *Inorganic Chemistry:* G. S. Applegate. *Organic Chemistry:* R. M. Hiley. Conjoint Board of England.—*First Examination: Chemistry and Physics*—G. H. Griffiths. *Second Examination: Anatomy and Physiology*—S. H. Kingston, E. R. Sircom, P. Sinnock, M. W. Morrison. *Final Examination: Medicine*—J. S. Avery,* C. S. Rivington. *Surgery*—F. Shingleton Smith,* M. R. O. Wilson, R. O. Bodman.* *Midwifery*—H. W. Goodden, R. E. Thomas, W. W. King, C. S. Rivington. *D.P.H. Lond.:* P. G. Stock, M.R.C.S., L.R.C.P. *L.S.A.: Surgery* (Section I.), *Medicine* (Section I.), *Forensic Medicine*—P. Moxey.

**Bristol Royal Infirmary.**—*Honorary Consulting Physician*—Henry Waldo, M.D., C.M. Aberd., M.R.C.P. *Honorary*

* Completes Examination.

*Consulting Surgeon*—A. W. Prichard, M.R.C.S. *Honorary Physician*—F. H. Edgeworth, M.B., B.C. Cantab., D.Sc. Lond. *Honorary Surgeon*—T. Carwardine, M.B., M.S. Lond., F.R.C.S. Eng. *Assistant Physician*—J. A. Nixon, M.B., B.C. Cantab., M.R.C.P. *Assistant Surgeon*—E. H. E. Stack, M.B., B.C. Cantab., F.R.C.S. Eng. *Pathologist, Bacteriologist, and Director of Clinical Laboratories*—I. Walker Hall, M.D., Ch.B. Vict. *House Physician*—A. L. Sheppard, M.B., B.S. Durh. *Senior Resident Officer*—A. R. Short, M.B., B.S., B.Sc. Lond. *Resident Obstetric Officer*—F. Rudge, M.R.C.S., L.R.C.P. *Casualty Officer*—A. J. Wright, M.R.C.S., L.R.C.P. *Junior House Surgeon*—L. Shingleton Smith, M.R.C.S., L.R.C.P.

**Health of Bath.**—At a meeting of the Sanitary Committee held on January 29th, the Medical Officer of Health (Dr. W. H. Symons), reported that during 1905 there were 985 births and 808 deaths registered in Bath. Estimating the population at 50,000, the birth-rate corresponded to 19.7 per 1,000, and the crude death-rate was 16.16 per 1,000. The corrected death-rate was 13 per 1,000, being the lowest rate recorded for Bath, except in the year 1903, when it was 12.3 per 1,000.

**Cardiff Infirmary.**—In spite of the £20,000 which has been expended during the last five years in order to make improvements in this institution, it will shortly have to make further enlargements for the accommodation of out-patients. Only 178 patients can now be received in the wards, and the necessity for increased accommodation is shown from the fact that early in this year 336 patients were awaiting admission. It is now decided to re-arrange the out-patient and nurses departments, the estimated cost of which is £7,000, towards which sum £1,000 has been offered by an anonymous donor provided the remainder is promised during the present year.

**The Royal West of England Sanatorium, Weston-super-Mare.**—During the past year 2,470 cases were admitted, the daily average number of patients being nearly 200. It has recently been decided to build an annexe to the institution, and this will be used by the nursing staff, and the wards which they at present occupy will give more accommodation to patients. It will be utilised for any infectious case should an outbreak arise in the sanatorium. The committee has received £900 from a legacy which will be devoted towards the building, and it is hoped that the additional sum required will be shortly forthcoming.

### Bristol Medico-Chirurgical Society.

### President.

JOHN DACRE.

### President-Elect.

JAMES TAYLOR.

### Honorary Secretary.

H. F. MOLE.

### Committee.

*Past Presidents.*
L. M. GRIFFITHS.
R. SHINGLETON SMITH, M.D., B.Sc. Lond.
NELSON C. DOBSON.
F. R. CROSS, M.B. Lond.
E. MARKHAM SKERRITT, M.D., B.S., B.A. Lond.
A. J. HARRISON, M.B. Lond.
ARTHUR W. PRICHARD.
J. E. SHAW, M.B., C.M. Ed.
R. ROXBURGH, M.B. Ed.
W. H. HARSANT.
D. S. DAVIES, M.D. Lond.
BARCLAY J. BARON, M.B., C.M. Edin.
G. MUNRO SMITH.
J. PAUL BUSH, C.M.G.
REGINALD EAGER, M.D. Lond.

J. MICHELL CLARKE, M.D., M.A. Cantab.
B. M. H. ROGERS, M.D., B.Ch., B.A. Oxon.
C. K. RUDGE.
JAMES SWAIN, M.D., M.S. Lond.
H. WALDO, M.D., C.M. Aberd.
P. WATSON WILLIAMS, M.D. Lond.

Medical Practitioners wishing to join the Society are requested to communicate with the Hon. Secretary, H. F. Mole, 19 Mortimer Road, Clifton, Bristol. The Annual Subscription is 21/-.

*Extracts from Journal By-laws.*

4.—The *Journal* shall be delivered free to Subscribers and also to Members of the Society whose subscriptions are not in arrear.

6.—The Annual Subscription to the *Journal* for those who pay before the 1st of July shall be 5/-, post free, or 6/- if paid after that date.

Communications intended for insertion in the *Journal* should be sent to the Editor, Dr. Shingleton Smith, Deepholm, Clifton Park, Clifton, Bristol.

Books for Review and Exchange Journals should be sent to the Assistant-Editor, Dr. P. Watson Williams, Medical Department, University College, Bristol.

Communications referring to the delivery of the *Journal* should be sent to the Editorial Secretary, Mr. James Taylor, 36 Alma Road, Clifton, Bristol, to whom Subscriptions are to be paid *in advance.*

Cases for Binding Vols. I.–XXIII. are now ready, and may be obtained, price 7*d.* each (postage 2*d.* extra), upon application to J. W. Arrowsmith, Quay Street, Bristol; or the *Journal* will be bound, including Case, for 1/3 per volume.

### Bristol Medico-Chirurgical Society.

### PAST PRESIDENTS.

1874—75. FREDERICK BRITTAN, M.D., B.A. Dub.
1875—76. ROBERT WILLIAM COE.
1876—77. SAMUEL MARTYN, M.D. Ed.
1877—78. AUGUSTIN PRICHARD, M.D. Berlin.
1878—79. HENRY EDWARD FRIPP, M.D. Würzburg.
1879—80. WILLIAM MICHELL CLARKE.
1880—81. JOSEPH GRIFFITHS SWAYNE, M.D. Lond.
1881—82. EDWARD LONG FOX, M.D. Oxon.
1882—83. JAMES GEORGE DAVEY, M.D. St. And.
1883—84. WILLIAM JOHNSTONE FYFFE, M.D., B.A. Dub.
1884—85. GEORGE FORSTER BURDER, M.D. Aberd.
1885—86. WILLIAM HENRY SPENCER, M.D., M.A. Cantab.
1886—87. FRANCIS POOLE LANSDOWN.
1887—88. LEMUEL MATTHEWS GRIFFITHS.
1888—89. ROBERT SHINGLETON SMITH, M.D., B.Sc. Lond.
1889—90. NELSON CONGREVE DOBSON.
1890—91. SAMUEL HENRY SWAYNE.
1891—92. FRANCIS RICHARDSON CROSS, M.B. Lond.
1892—93. EDWARD MARKHAM SKERRITT, M.D., B.S., B.A. Lond.
1893—94. JAMES GREIG SMITH, M.B., C.M., M.A. Aberd., F.R.S.E.
1894—95. ALFRED JAMES HARRISON, M.B. Lond.
1895—96. ARTHUR WILLIAM PRICHARD.
1896—97. ALFRED EDWARD AUST LAWRENCE, M.D., C.M. Aberd.
1897—98. JOHN EDWARD SHAW, M.B., C.M. Ed.
1898—99. ROBERT ROXBURGH, M.B. Ed.
1899—00. WILLIAM HENRY HARSANT.
1900—01. DAVID SAMUEL DAVIES, M.D. Lond.
1901—02. BARCLAY JOSIAH BARON, M.B., C.M. Ed.
1902—03. GEORGE MUNRO SMITH.
1903—04. JAMES PAUL BUSH, C.M.G.
1904—05. REGINALD EAGER, M.D. Lond.

1906.

---

### LIST OF SUBSCRIBERS

TO THE

### Bristol Medico-Chirurgical Journal.

---

*Those marked * are Members of the Society.*

| | |
|---|---|
| Ackland, J. McKno ... ... ... ... ... ... | 24 Southernhay, Exeter. |
| *Ackland, W. R. ... ... ... ... ... ... ... | 5 Rodney Place, Clifton. |
| Adye, W. J. A. ... ... ... ... ... ... ... .. | Church House, Bradford, Wilts. |
| Aldous, George F.... ... ... ... ... ... ... | Charlton House, Mannamead, Plymouth. |
| Aldridge, Charles, M.D., C.M. Aberd.... ... | Plympton. |
| *Alexander, D.A., M.B., Ch.B. Vict.... ... ... | 30 Berkeley Square, Clifton. |
| Ambrose, J., M.B., C.M. Aberd. ... ... ... ... | 138 Fishponds Road, Eastville, Bristol. |
| Aveline, H. T. S. ... ... ... ... ... ... ... | Somerset Asylum, Taunton. |
| *Ballance, H. Stanley, M.D. Lond. ... ... | 4 Royal Terrace, Weston-super-Mare. |
| Barber, M. C. ... ... ... ... ... ... ... | Glenavon House, Staple Hill, Bristol. |
| Baretti, T. G. L'Enardi ... ... ... ... ... | 49 Royal York Crescent, Clifton. |
| *Barker, G. H., M.B., B.Sc. Lond. ... ... ... | 124 Redland Road, Bristol |
| *Baron, Barclay J., M.B., C.M. Ed.... ... ... | 16 Whiteladies Road, Clifton. |
| *Basset, Walter ... ... ... ... ... ... ... | 24 Stow Hill, Newport, Mon. |
| Batten, Rayner W., M.D. Lond. ... ... ... | 1 Brunswick Square, Gloucester. |
| Batten, W. Smith... ... ... ... ... ... ... | West Hill, Calne. |
| Bayer Co., The ... ... ... ... ... ... ... | 19 St. Dunstan's Hill, London, E.C. |
| *Beddoe, John, M.D. Ed., B.A. Lond., F.R.S. | The Chantry, Bradford-on-Avon. |
| Begg, C., M.B., C.M. Edin.... ... ... ... ... | 62 Pulteney Street, Bath. |

### LIST OF SUBSCRIBERS.

| | |
|---|---|
| Bennett, W. S. | Escot, Penzance. |
| Bernard, C. | 2 Spencer Terrace, Fishponds. |
| *Berry, F. C., M.D., B.Ch., B.A. Dub. | Burnham, Somerset. |
| Berry, James, M.B., B.S. Lond. | 21 Wimpole Street, London, W. |
| *Beverley, K. H. | Children's Hospital, Bristol. |
| Biamonti, Sebastiano | 27 Via Balbi, Genoa, Italy. |
| *Blacker, A. E. | 20 Victoria Square, Clifton. |
| *Blagg, Arthur F., M.D. Durh. | 28 Caledonia Place, Clifton. |
| Board, J. H. | 8 Caledonia Place, Clifton. |
| Bodman, Hervey, M.D. Lond. | 3 Whiteladies Road, Clifton. |
| Boyce, James G. | The Chipping, Wotton-under-Edge. |
| Boyd, Geo. S. J. | 19 St. George's Square, Stamford, Lincs. |
| *Brasher, Charles W. J. | 128 Cotham Brow, Bristol. |
| *Bristowe, H. C., M.D. Lond. | Wrington, Somerset. |
| Brown, G. Arthur | Tredegar. |
| Brown, William, M.D., C.M. Glasg. | Park View, Fishponds, Gloucestershire. |
| *Bullen, F. St. John | 12 Pembroke Road, Clifton. |
| *Bunbury, E. G. | 13 Dowry Square, Hotwells. |
| Burroughs, E. F. H. | 3 Elgin Park, Bristol. |
| *Bush, J. Paul, C.M.G. | Vyvyan House, Clifton Park, Clifton. |

| | |
|---|---|
| *Campbell-Horsfall, C.E., M.B., Ch.B. (Vict.) | Sisson Lodge, Clevedon. |
| *Carter, T. M. | 28 Victoria Square, Clifton. |
| *Carwardine, Thomas, M.B., M.S. Lond. | 16 Victoria Square, Clifton. |
| *Cave, Edward J., M.D. Lond. | 20 Circus, Bath. |
| Chung, W. C. | Imperial Chinese Hospital, Chinanfu, Shan-tung, China. |
| Chute, Henry M. | King William's Town, S. Africa. |
| *Clarke, John Michell, M.D., M.A. Cantab. | 28 Pembroke Road, Clifton. |
| Cobbledick, A. S., M.D., B.S. Lond. | 402 Brixton Road, London, S.W. |
| Cochran, Alexander, M.B., C.M. Glasg. | Bath Road, New Brislington, Bristol. |
| *Collinson, T. A. | 11 Zetland Road, Redland, Bristol. |
| *Cook, Henri, M.D., C.M. Aberd. | 1 Dean Lane, Southville, Bristol. |
| *Coombs, Carey, M.D. Lond. | 11 Henleaze Road, Westbury-on-Trym. |
| *Corfield, C. | 18 Elmdale Road, Tyndall's Park, Clifton. |
| *Corrigan, W. J. | Cloughmore, Splott Avenue, Cardiff. |
| Cossham, W. R., M.D., C.M. Aberd. | Cirencester. |
| Cotton, William, M.B., C.M., M.A. Ed. | 231 Gloucester Road, Bristol. |
| Coulter, R. J., M.B. Dub. | 11 Clytha Park Road, Newport, Mon. |
| Cousins, J. Ward, M.D. Lond. | Kent Road, Southsea. |
| Cowan, F. S. | 27 Queen Square, Bath. |
| Craddock, Samuel | South Lyncombe, Bath. |
| Cridland, A. B. | 52 Waterloo Road, Wolverhampton. |
| Crisp, J. Ellis | Alexander House, Corsham. |
| *Cross, F. Richardson, M.B. Lond. | Worcester House, Clifton. |
| *Crossman, F. W. | Hambrook, Gloucestershire. |
| *Crouch, C. Percival, M.B. Lond. | 1 Royal Terrace, Weston-super-Mare. |
| Culross, James, M.B., C.M., M.A. Glasg. | 66 Queen Street, Newton Abbot. |

| | |
|---|---|
| *Dacre, John | 14 Eaton Crescent, Clifton. |
| Dalby, Augustus W. | Argyll House, Frome. |
| *Davies, D. S., M.D. Lond., D.P.H. Cantab. | 60 Oakfield Road, Clifton. |

### LIST OF SUBSCRIBERS.

| | |
|---|---|
| Davy, Henry, M.D. Lond. ... ... ... ... ... ... | Southernhay House, Exeter. |
| Dening, Edwin ... ... ... ... ... ... ... | Manor House, Stow-on-the-Wold. |
| Devine, H. ... ... ... ... ... ... ... ... | London County Asylum, Cane Hill, Purley, Surrey. |
| Dickie, James Steel, M.D., C.M. Aberd. ... | Boscombe Park, Bournemouth. |
| *Dobson, Nelson C. ... ... ... ... ... ... | Avonmore, Sneyd Park. |
| Dunbar, Eliza L. Walker, M.D. Zürich ... | 9 Oakfield Road, Clifton. |
| Dymoke, F. ... ... ... ... ... ... ... ... | 21 Cotham Road, Bristol. |
| Eadon, W. F. Bailey ... ... ... ... ... ... | Hambrook, Gloucestershire. |
| *Eager, Reginald, M.D. Lond. ... ... ... ... | Northwoods, Winterbourne. |
| *Eager, W. R. ... ... ... ... ... ... ... ... | Northwoods, Winterbourne. |
| Eberle, Emily, M.A., M.B., B.Ch. Dub. ... | 17 Oakfield Road, Clifton. |
| *Edgeworth, F. H., M.B., B.C., B.A. Cantab., D.Sc. Lond. ... ... ... ... ... ... ... ... | 35 Pembroke Road, Clifton. |
| Edwards, E. S. ... ... ... ... ... ... ... | Empingham, Stamford. |
| Elder, William ... ... ... ... ... ... ... | 4 Trelawney Road, Bristol. |
| Elliot Bros. Ltd. ... ... ... ... ... ... | *Australian Pharmaceutical Notes & News.* O'Connell Street, Sydney, N.S.W. |
| *Elliott, Christopher, M.D., B.A. Dub. ... | 3 Beaufort Road, Clifton. |
| Elliott, F. Percy, M.B., C.M. Aberd. ... ... | 113 Grove Road, Walthamstow, London. N.E. |
| Ellis, W. McD., M.D. Brux. ... ... ... ... | 25 The Circus, Bath. |
| Evans, T. G. C. ... ... ... ... ... ... ... | Budleigh Salterton, Devon. |
| Every-Clayton, L. E. V., M.D. Lond. ... ... | Rosslyn, Clevedon, Som.erset |
| Fairbanks, W., M.D., C.M. Ed. ... ... ... ... | Wells, Somerset. |
| *Ferguson, R. S., B.A. Durh., M.B. C.M. Edin. | Elm Grove, Calne, Wilts. |
| *Firth, J. L., M.D., M.S. Lond. ... ... ... ... | Victoria Square, Clifton. |
| Fisher, Theodore, M.D. Lond. ... ... ... | North Eastern Hospital, London. |
| *Flemming, A. L. ... ... ... ... ... ... ... | 34 Alma Road, Clifton. |
| *Flemming, C. E. S. ... ... ... ... ... ... | Manvers House, Bradford-on-Avon. |
| *Fletcher, J., M.D., C.M., D.P.H. Aberd. ... | Ham Green, Somerset. |
| Flood, P. ... ... ... ... ... ... ... ... ... | 318 Stapleton Road, Bristol. |
| *Fortescue-Brickdale, J. M., M.A., M.D., B.Ch. Oxon.... ... ... ... ... ... ... ... | 52 Pembroke Road, Clifton. |
| Forty, D. H. ... ... ... ... ... ... ... ... | Wotton-under-Edge. |
| Foss, E. V. ... ... ... ... ... ... ... ... | Babbacombe House, Ashton Road, Bedminster, Bristol. |
| Fowler, O. H. ... ... ... ... ... ... ... ... | Cirencester. |
| Fox, F. F. ... ... ... ... ... ... ... ... ... | Yate House, Chipping Sodbury. |
| Fox, T. Colcott, M.B. Lond., B.A. Cantab.... | 14 Harley Street, London, W. |
| *Fraser, Forbes ... ... ... ... ... ... ... | 2 The Circus, Bath. |
| Frazer-Toovey, T. E. ... ... ... ... ... ... | Balfour House, Downs Road, Clapton, London, N.E. |
| Freeman, J., M.D. Brux. ... ... ... ... ... | Glencairn Villa, Queen's Road, Clifton. |
| Garland, E. B., M.B., C.M. Edin. ... ... ... | 104*a* Hampton Road, Redland. |
| Genge, T. T. ... ... ... ... ... ... ... ... | 21 Whiteladies Road, Bristol. |
| *Gerrish, D. S. ... ... ... ... ... ... ... ... | 322 Church Road, St. George, Bristol. |

### LIST OF SUBSCRIBERS.

| | |
|---|---|
| Goss, T. Biddulph | 1 The Circus, Bath. |
| Grace, Alfred | Chipping Sodbury. |
| Grace, Edward Mills | Thornbury, Gloucestershire |
| Gratte, C. Brooke | 183 Commercial Road, Newport, Mon. |
| *Green, T. A., M.D., C.M. Edin. | 33*a* Whiteladies Road, Clifton. |
| *Greer, W. J. | 2 Chepstow Road, Newport, Mon. |
| Griffiths, Cornelius A. | 6 Windsor Place, Cardiff. |
| *Griffiths, Lt.-Col. G.S. | 11 Vyvyan Terrace, Clifton. |
| *Griffiths, J. S. | 20 Redland Park, Bristol. |
| *Griffiths, L. M. | 11 Pembroke Road, Clifton. |
| *Groves, E. W. H., M.B., B.Sc. Lond. | 16 Richmond Hill, Clifton. |

| | |
|---|---|
| Hadwen, W. R., M.D. St. And. | 35 Brunswick Square, Gloucester. |
| *Hailes, Clements D. G., M.D., C.M. Ed. | 27 Alma Road, Clifton. |
| *Haines, A. H. | 8 Cotham Road, Bristol. |
| *Hall, E. G., M.B. Lond. | 53 Hampton Road, Redland, Bristol. |
| Handfield-Jones, Montagu, M.D. Lond. | 35 Cavendish Square, London, W. |
| *Harris, H. Elwin, B.A., M.B. Cantab. | 13 Lansdown Place, Clifton. |
| *Harrison, A. J., M.B. Lond. | Guthrie Road, Clifton. |
| *Harsant, W. H. | Tower House, Clifton. |
| Hartnell, E. B. | Thornbury, R.S.O., Glos. |
| *Harvey, Alfred, M.B. Lond. | Westbury-on-Trym. |
| Hayman, Alfred G. | Hapsford House, near Frome. |
| *Hayman, C. A., M.D. St. And. | Kingston Villa, Richmond Hill, Clifton. |
| *Heaven, John C., D.P.H. Lond. | 17 Whiteladies Road, Bristol. |
| *Herapath, C. K. C. | Cotham Park, Bristol. |
| Hill, Hedley | 1 Redcliff Hill, Bristol. |
| *Hill, Walter J. | The Brow, Clevedon. |
| Hoffman, A. W. W. | 30 The Parade, Leamington. |
| *Holmes, Thomas E., M.A., M.B., B.C. Cantab. | Rosendal, Redland Road, Bristol. |
| Hospital, Bristol General (*Sec.*, W. Thwaites). | |
| Hospital, Taunton and Somerset (*House-Surgeon*, W. Drake). | |
| Huggard W. R., M.D., M.Ch., M.A. Roy. Univ. | Davos-Platz, Switzerland. |

| | |
|---|---|
| Imlay, G. A., M.B., C.M. Aberd. | 1 Cotham Park, Bristol. |
| Ingle, C. Durban | Somerton, Somerset. |

| | |
|---|---|
| Jamison, A., M.B., B.Ch., M.A. Roy. Univ. I. | 66 Woodstock Road, Belfast. |
| Jones, Evan | Ty-mawr, Aberdare. |
| *Jones, E. J. Trevor, M.D. Brux. | Aberdare, Glamorganshire. |
| Jones, H. Macnaughton, M.D., M.Ch. Roy. U.I. | 131 Harley Street, London, W. |

### LIST OF SUBSCRIBERS.

| | |
|---|---|
| *Keir, J. C., M.B., Ch.B. Ed. | The Limes, Melksham. |
| *Kemm, F. St. J.... | Worle. |
| *Kendall, H. W. | 1 Burlington Road, Redland. |
| *Kyle, H. G., M.B., B.Ch. Oxon. | 31 Westbury Road, Westbury-on-Trym. |

| | |
|---|---|
| *Lace, F. | 1 Paragon, Bath. |
| Lancaster, E. Le Cronier, B.A., M.B., B.Ch. Oxon. | Winchester House, Swansea. |
| *Lansdown, R. G. P., M.B., B.S. Dur. | 39 Oakfield Road, Clifton. |
| Lawrie, J. Macpherson, M.D., C.M. Glasg.... | Greenhill, Weymouth. |
| Le Soudier, H. | 174 et 176 Boulevard St. Germain, Paris. |
| *Lees, E. Leonard, M.D., C.M. Ed. ... | 1 Chesterfield Place, Clifton. |
| Leigh, W. W. ... | Treharris, Glamorganshire. |
| *Leonard, R. C., M.D. Ed. ... | 134 Coronation Road, Bristol. |
| Lowe, T. Pagan | 16 Circus, Bath. |
| *Lucas, J. J. S., M.D. Lond. ... | 186 Wells Road, Totterdown. |
| Lucy, Reginald H. | 9 The Crescent, Plymouth. |

| | |
|---|---|
| MacCarthy, Ffennell, M.D. Dub. | 21 Alexandra Road, Westbury Park. |
| *McCrea, P. W., M.D., B.Ch., B.A.O., B.A. Dub. | 19 Portland Square, Bristol. |
| MacDonald, P. W., M.D., C.M. Aberd. ... | Herrison, Dorchester. |
| Mackinnon, Charles, M.B., C.M., M.A. Glasg. | Davaar, Cirencester. |
| Maclean, Ewen J., M.D., C.M. Ed.... | 12 Park Place, Cardiff. |
| Maclean, J. Campbell, M.B., C.M. Ed.... | Swindon House, Swindon, Wiltshire. |
| Macready, J. ... | 42 Devonshire Street, Portland Place, London, W. |
| McWatters, J. C. | Almondsbury. |
| Marsh, E. H. ... | Long Preston, Yorks. |
| Marsh, O. E. Bulwer ... | Parkdale, Newport, Mon. |
| Marshall, Arthur L., M.B., B.A. Cantab ... | Thornleigh, Upper Clapton, London, N.E. |
| *Martin, E. F., M.B. Ed. | 7 Royal Terrace, Weston-super-Mare. |
| *Martin, R C. ... | 3 Clifton Vale, Clifton. |
| *Martyn, G. J. King, M D., B.C., B.A. Cantab. ... | 12 Gay Street, Bath. |
| Mason, S. B. | 12 Park Terrace, Pontypool. |
| Mason, William | Sedgemoor Cottage, St. Austell, Cornwall. |
| Matthews, Charles E., M.D. Dur. | 6 Hickman Road, Penarth. |
| Matthews, H. E. ... | The Mall Pharmacy, Clifton. |
| Meredith, John, M.D. Ed. ... | Wellington, Somerset. |
| Millard, W. J. Kelson, M.D. St. And. ... | 7 Bayshill Villas, Cheltenham. |
| Mitchell, Trafford, M.D. | Gorseinon, R.S.O., Glamorganshire. |
| *Mole, Harold F. ... | 19 Mortimer Road, Clifton, Bristol. |
| Monckton, William | Portishead. |
| *Moore, L. A. ... | 45 Ashley Road, Bristol. |

| | |
|---|---|
| MOORES, DE LA HEY ... ... ... ... .. ... | 6 West Mall, Clifton. |
| MORGAN, ALBERT T., M.D. Brux. ... ... ... | 89 Chesterfield Road, St. Andrew's Park Bristol. |
| *MORGAN, T. WHITWORTH S. ... ... ... ... | The Laurels, Timsbury, Bath. |
| *MORTON, C. A. ... ... ... ... ... ... ... ... ... | 14 Vyvyan Terrace, Clifton. |
| *MORTON, W. B., M.D. Lond. ... ... ... ... | Brislington House, Brislington. |
| MUNDEN, CHARLES ... ... ... ... ... ... ... | Ilminster. |
| *MYLES, G T. ... ... ... ... ... ... ... ... ... | 147 Whiteladies Road, Bristol. |

| | |
|---|---|
| *NEILD, NEWMAN, M.B., B.Ch. Vict. ... ... ... | 9 Richmond Hill, Clifton. |
| *NEVILL, W. N., M.D., B.Ch., B.A. Dub. ... ... | 12 Acraman's Road, Southville, Bristol. |
| *NEWMAN, CHARLES ... ... ... ... ... ... ... | 156 Cheltenham Road, Bristol. |
| *NEWNHAM, W. H. C., M.B., M.A. Cantab. ... | 3 Lansdown Place, Victoria Square, Clifton. |
| NICHOLSON, T. D., M.D. Ed. ... ... ... ... | 2 Whiteladies Road, Clifton. |
| *NIXON, J. A., B.A., M.B., B.C. Cantab. ... ... | 18 West Mall, Clifton. |
| NORMAN, J. H. ... ... ... ... ... ... ... ... | Goodleigh, Winkleigh, Devonshire. |

| | |
|---|---|
| O'BRIEN, CORNELIUS ... ... ... ... ... ... ... | Devon House, Whitehall, Bristol. |
| ODELL, WILLIAM, M.D. Durh. ... ... ... ... | Ferndale, Torquay. |
| *OGILVY, ALEXANDER, M.D., B.Ch., B.A. Dub. | Montrose House, Queen's Road, Clifton. |
| OPENSHAW, E. H. ... ... ... ... ... ... ... ... | Cheddar. |
| *ORMEROD, H. LAWRENCE, M.B., B.Ch., B.A.O., R.U.I. ... ... ... ... ... ... ... ... ... | 2 Henleaze Road, Westbury Park. |

| | |
|---|---|
| PAGE, G. SHEPLEY ... ... ... ... ... ... ... | 78 Old Market Street, Bristol. |
| PAPILLON, J. W. ... ... ... ... ... ... ... | Brent Knoll, Bridgwater. |
| *PARKER, GEORGE, M.D., M.A. Cantab. ... ... | 14 Pembroke Road, Clifton. |
| PARRY, J. H. ... ... ... ... ... ... ... ... | Ashley Road, Bristol. |
| *PARSONS, H. F. ... ... ... ... ... ... ... ... | The Heath, Stoke Bishop. |
| PARSONS, J. H., M.B., B.S., D.Sc. Lond. ... ... | 27 Wimpole Street, London, W. |
| PATERSON, DONALD ROSE, M.D., C.M. Ed. ... | 18 Windsor Place, Cardiff. |
| PEAKE, ARTHUR W. ... ... ... ... ... ... ... | 34 Gloucester Road, Bishopston, Bristol. |
| PEAKE, G. ARTHUR ... ... ... ... ... ... ... | Rodney Place, Cheltenham. |
| PEARSE, E. M. ... ... ... ... ... ... ... ... | 2 Varley Terrace, Liskeard. |
| PECK, Lt.-Col. F.S., I.M.S. ... ... ... ... | 6 Harington Street, Calcutta. |
| PHILLIPS, C. M., M.D. Brux. ... ... ... ... | Ravenslea, Kensington Hill, Brislington. |
| PHILLIPS, EDWARD PICTON ... ... ... ... ... | High Street, Haverfordwest. |
| *PICKERING, C. F. ... ... ... ... ... ... ... | 13 Berkeley Square, Bristol. |
| POWELL, J. J., M.D. Lond. ... ... ... ... ... | 2 Gloucester Road, Bishopston, Bristol. |
| *POWELL, L. W. ... ... ... ... ... ... ... ... | Two Mile Hill Road, Kingswood, Bristol |
| POWELL, WILLIAM, M.B. Lond. ... ... ... ... | Hill Garden, Torquay |
| PRICE, D. T., M.B. Lond. ... ... ... ... ... | Castle Cary, Somerset. |

### LIST OF SUBSCRIBERS.

| | |
|---|---|
| *PRICHARD, ARTHUR W. | 6 Rodney Place, Clifton. |
| *PROWSE, ARTHUR B., M.D. Lond. | 5 Lansdown Place, Clifton. |
| PROWSE, W. B. | 11 St. George's Place, Brighton. |
| PRUEN, S. T., M.D. Dur. | Sherborne Lodge, Cheltenham. |
| RANDOLPH, CHARLES | St. Michael's, Milverton, Somerset. |
| *RATTRAY, J. M., M.D., C.M., M.A. Aberd. | Frome. |
| *RAYNER, D. C. | 9 Lansdown Place, Clifton, Bristol. |
| REDWOOD, T. HALL, M.D., C.M., M.A. Dur. | The Lawn, Rhymney, Monmouthshire. |
| RENDALL, JOHN | Forestside, Lymington. |
| *REW, G. R. | Children's Hospital, Bristol. |
| RICHARDS, D. T. | The Meadows, Risca, Mon. |
| *RICHARDSON, A. W., M.B. Lond. | Bristol General Hospital. |
| RIDDELL, ANDREW W. | 91 Rowson Street, New Brighton, Cheshire. |
| RITCHIE, THOMAS P. | 3 Redcliff Parade West. |
| ROBERTS, FREDERICK T., M.D., B.Sc. Lond. | 102 Harley Street, London, W. |
| RODGERS, JOHN W., M.B., C.M. Ed. | Redfield, Gloucestershire. |
| *ROGERS, B. M. H., M.D., B.Ch., B.A. Oxon. | 1 Victoria Square, Clifton. |
| *ROLFE, R., B.A., M.B., B.C. Camb. | Roseneath, Avonmouth. |
| *ROSE, F. H. | 13 Westfield Park, Clifton. |
| *ROSSITER, GEORGE F., M.B. Lond. | Cairo Lodge, Weston-super-Mare. |
| *ROUÉ, W. BARRETT, M.D., M.S. Dur. | 2 Buckingham Place, Clifton. |
| *ROXBURGH, ROBERT, M.B. Ed. | 1 Victoria Buildings, Weston-super-Mare. |
| *RUDGE, C. KING | 145 Whiteladies Road, Bristol. |
| RUFFER, M. ARMAND, M.D., B.S., M.B. Oxon | Ramleh, Alexandria, Egypt. |
| RUTHERFOORD, H. T., M.A., M.D. Camb. | Salisbury House, Taunton. |
| SCOTT, RICHARD J. H. | 28 Circus, Bath. |
| SEWARD, W. J., M.B. Lond. | Colney Hatch Asylum, New Southgate, London, N. |
| *SHAW, J. E., M.B., C.M. Ed. | 23 Caledonia Place, Clifton. |
| SHAW-MACKENZIE, JOHN A., M.D. Lond. | 42 Green Street, Park Lane, London, W. |
| *SHEPPARD, E. J. | 8 Richmond Hill, Clifton. |
| *SHOOLBRED, W. A. | St. Ann's, Chepstow. |
| *SHORT, A. R., M.D., B.S., B.Sc. Lond. | Bristol Royal Infirmary. |
| *SIEVEKING, A. R. | 18 Blenheim Road, Redland. |
| SIMONS, R. J. | Cae Court, Bridgend. |
| SKELTON, HENRY, M.D. St. And. | Downend, Gloucestershire. |
| *SKERRITT, E. MARKHAM, M.D., B.S., B.A. Lond. | Ivor House, Durdham Park, Bristol. |
| *SMITH, G. COCKBURN, M.D. Brux. | 14 South Road, Newton Abbot. |
| *SMITH, G. MUNRO | 18 Apsley Road, Clifton. |
| SMITH, REV. REGINALD, M.A. Cantab. | The Vicarage, Walpole St. Andrew Norfolk. |
| *SMITH, R. SHINGLETON, M.D., B.Sc. Lond. | Clifton Park, Clifton. |
| SMITH, W., M.B. Lond. | Brabourne, Ashford, Kent. |
| SMITH, W. ALEXANDER, M.B., M.A. Oxon. | Newport, Essex. |
| *SMITH, W. A. L., M.A., M.B., B.C. Cantab. | 8 Chamberlain Street, Wells. |
| SMYTH, H. J. | New Road, South Molton. |

SOCIETY, CARDIFF MEDICAL (*Hon. Lib.*, Queen Street, Cardiff).

### LIST OF SUBSCRIBERS.

| | |
|---|---|
| SOCIETY, MANCHESTER MEDICAL ... ... ... | Medical Society's Library, The Owens College, Manchester. |
| SOCIETY, PLYMOUTH MEDICAL (*Hon. Lib.*, J. ELLIOTT SQUARE). | |
| *STACK, E. H. E., B.A., M.B., B.C. Cantab. ... | 10 Whiteladies Road, Clifton. |
| STAMP, W. D. ... ... ... ... ... ... ... ... | Ridgeway House, Plympton. |
| STAPLE, J. D. ... ... ... ... ... ... ... ... | Clevedon Villa, Cheltenham Road, Bristol. |
| STATHAM, R. WHITESIDE ... ... ... ... ... | Cheddar, Somersetshire. |
| *STEELE, CHARLES, M.D. Dur. ... ... ... ... | 17 Richmond Hill, Clifton. |
| *STEELE, W. H., Lt.-Col., M.D. Dub. ... ... | 18 Mortimer Road, Clifton. |
| STEVENS, W. H. ... ... ... ... ... ... ... | 26 Zetland Road, Bristol. |
| *STEWART, J., B.A., R.U.I. ... ... ... ... ... | Dunmurry, Sneyd Park, Bristol. |
| STOCK, P. G. ... ... ... ... ... ... ... ... | Box 1049, Johannesburg, South Africa. |
| *STOCK, W. S. V., M.B., B.S. Lond. ... ... ... | 4 Leicester Place, Clifton. |
| STODDART, F. WALLIS ... ... ... ... ... ... | Southey House, College Green, Bristol. |
| *SWAIN, JAMES, M.D., M.S. Lond. ... ... ... | 4 Victoria Square, Clifton. |
| *SWAYNE, W. CARLESS, M.D. Lond. ... ... ... | St. Paul's Road, Clifton. |
| *SYMES, J. O., M.D. Lond. ... ... ... ... ... | 71 Pembroke Road, Clifton. |
| SYMONDS, H. P. ... ... ... ... ... ... ... | 35 Beaumont Street, Oxford. |
| SYMONS, JOHN ... ... ... ... ... ... ... ... | Buriton House, Penzance. |

| | |
|---|---|
| TAYLOR, C. BELL, M.D. Edin. ... ... ... ... | 9 Park Row, Nottingham. |
| *TAYLOR, C. J. ... ... ... ... ... ... ... ... ... | 54 Apsley Road, Clifton. |
| *TAYLOR, JAMES ... ... ... ... ... ... ... ... | 36 Alma Road, Clifton. |
| *TEMPLE, G. H., M.B., C.M. Ed. ... ... ... | Weston-super-Mare. |
| THOMAS, A. GARROD, M.D., C.M. Ed. ... ... | Clytha Park, Newport, Mon. |
| THOMAS, J. LYNN ... ... ... ... ... ... ... ... | 21 Windsor Place, Cardiff. |
| *THOMAS, J. M. M. ... ... ... ... ... ... ... | 5 Colston Parade, Redcliffe, Bristol. |
| THOMAS, RAGLAN, M.D. Lond. ... ... ... ... | 34 West Southernhay, Exeter. |
| THOMSON, EUSTACE B., M.D., C.M. Glasg. ... | Albany Place, Plymouth. |
| TIVY, W. J. ... ... ... ... ... ... ... ... ... | 5 Victoria Square, Clifton. |
| TOSSWILL, LOUIS H., M.B., B.A. Cantab.... ... | 1 West Southernhay, Exeter. |
| TREVITHICK, EDGAR, M.A., M.D. Cantab. ... | 24 Promenade, Cheltenham. |
| TYLEY, R. P., M.D. St. And.... ... ... ... ... | Wedmore. |

| | |
|---|---|
| VACHELL, HERBERT R., M.D., C.M. Aberd. ... | 18 Newport Road, Cardiff. |
| VEREKER, R. H. ... ... ... ... ... ... ... | Eastleigh, Curry Rivel, Somerset. |

| | |
|---|---|
| *WALDO, HENRY, M.D., C.M. Aberd. ... ... ... | 19 Pembroke Road, Clifton. |
| *WALKER, CYRIL H., M.B., B.A. Cantab. ... ... | 8 Oakfield Road, Clifton. |
| WALLACE, REV. CANON, M.A. Oxon. ... ... ... | 3 Gloucester Row, Clifton. |
| WALLACE, J. T., M.B., B.Ch. R.U.I.... ... ... | 48 Coronation Road, Bristol. |
| *WALLACE, JOHN ... ... ... ... ... ... ... | 1 Oriel Terrace, Weston-super-Mare. |
| WALTERS, C. F. ... ... ... ... ... ... ... ... | 13 Tothill Avenue, Plymouth. |
| WARD, J. L. W.... ... ... ... ... ... ... ... ... | Victoria Street, Merthyr Tydvil. |
| WEATHERLY, LIONEL A., M.D., C.M. Aberd. | Bailbrook House, Bath. |
| WEBBER, WILLIAM W. ... ... ... ... ... ... | Crewkerne. |
| *WESTCOTT, W. G. ... ... ... ... ... ... ... | H.M. Dockyard, Malta. |
| *WHICHER, A. H. ... ... ... ... ... ... ... | 115 Queen's Road, Clifton. |

| | |
|---|---|
| White, C. R. ... ... ... ... ... ... ... ... | Nixon Villa, Merthyr Vale, Glamorgan. |
| White, J. W. ... ... ... ... ... ... ... ... | Orchard House, Nailsea. |
| *White, W. Percy ... ... ... ... ... ... ... | College Green, Bristol. |
| *Whitmore, F. C. ... ... ... ... ... ... ... | 497 Fishponds Road, Bristol. |
| Wigan, C. A., M.D. Dur. ... ... ... ... ... | Portishead. |
| Wigmore, J., M.D. Roy. Univ. I. ... ... ... | 20 Green Park, Bath. |
| Wilcox, Henry, M.B. Aberd. ... ... ... ... | Fleet, Hampshire. |
| Willcox, R. Lewis ... ... ... ... ... ... | Warminster. |
| Willett, George G. D. ... ... ... ... ... | Keynsham, Somerset. |
| *Williams, E. C., M.B., B.C., B.A. Cantab. ... | 9 Whatley Road, Clifton. |
| Williams, H. Egerton ... ... ... ... ... | The Mount, Newport, Mon. |
| *Williams, P. Watson, M.D. Lond.... ... ... | 4 Clifton Park, Clifton. |
| *Williams, W. Roger ... ... ... ... ... ... | Beaufort House, Clifton Down, Clifton |
| Willis, W. M.... ... ... ... ... ... ... ... | 7 Regent Street, Nottingham. |
| *Wills, W. K., M.B., B.C. Camb. ... ... ... | 19 Whiteladies Road, Clifton. |
| Wilson, E. T., M.D. Oxon.... ... ... ... ... | Westol, Cheltenham. |
| *Windsor-Aubrey, H. W. ... ... ... ... ... | Coronation Road, Bristol. |
| Woodhead, G. Sims, M.D., C.M. Ed. ... ... | Pathological Laboratory, New Museum, Cambridge. |
| Woollcombe, Walter L. ... ... ... ... ... | 16 The Crescent, Plymouth. |

---

**With Plates containing 93 Coloured and Stereoscopic Figures, 209 Illustrations in the Text, and a STEREOSCOPE complete, 17/6 net, including Stereoscope.**

**BY P. WATSON WILLIAMS, M.D. (LOND.),**

*Physician in Charge of Throat Department, Bristol Royal Infirmary; and to the Bristol Institute for the Deaf and Dumb.*

DISEASES OF THE

